# The ion microscope as a tool for quantitative measurements in the extreme ultraviolet

**DOI:** 10.1038/srep21556

**Published:** 2016-02-12

**Authors:** N. Tsatrafyllis, B. Bergues, H. Schröder, L. Veisz, E. Skantzakis, D. Gray, B. Bodi, S. Kuhn, G. D. Tsakiris, D. Charalambidis, P. Tzallas

**Affiliations:** 1 Foundation for Research and Technology - Hellas, Institute of Electronic Structure & Laser, PO Box 1527, GR71110 Heraklion (Crete), Greece; 2 Department of Physics, University of Crete, PO Box 2208, GR71003 Heraklion (Crete), Greece; 3 Max-Planck-Institut für Quantenoptik, D-85748 Garching, Germany; 4 Department of Physics, Umeå University, SE-901 87 Umeå, Sweden; 5 Wigner Research Center for Physics, 1121 Budapest, Hungary; 6 ELI Attosecond Light Pulse Source, ELI-Hu Kft., Dugonics ter 13, 6720 Szeged, Hungary

## Abstract

We demonstrate a tool for quantitative measurements in the extreme ultraviolet (EUV) spectral region measuring spatially resolved atomic ionization products at the focus of an EUV beam. The ionizing radiation is a comb of the 11^th^–15^th^ harmonics of a Ti:Sapphire femtosecond laser beam produced in a Xenon gas jet. The spatial ion distribution at the focus of the harmonics is recorded using an ion microscope. Spatially resolved single- and two-photon ionization products of Argon and Helium are observed. From such ion distributions single- and two-photon generalized cross sections can be extracted by a self-calibrating method. The observation of spatially resolved two-EUV-photon ionization constitutes an initial step towards future single-shot temporal characterization of attosecond pulses.

Laser driven higher order harmonic generation sources have been producing for quite a while Extreme Ultra Violet (EUV) pulses, which upon tight focusing reach intensities sufficiently high to induce observable two-EUV-photon processes. Thus, two-EUV-photon ionization has been observed using single harmonics, i.e. relatively long EUV-pulses^1^,^2^,^3^,^4^,^5^, harmonic combs, i.e. trains of EUV attosecond pulses^6^,^7^,^8^ as well as broadband coherent EUV continua, i.e. isolated pulses with durations of the order of 1 *fs* or shorter^9^,^10^. Two-EUV-photon processes have been exploited in EUV pulse metrology by means of second order autocorrelation measurements for isolated harmonics^11^, attosecond pulse trains^12^,^13^,^14^,^15^, broad band coherent EUV continua^9^,^10^ as well as recently demonstrated time resolved vacuum ultraviolet (VUV) and EUV spectroscopy^16^,^17^ and EUV-pump-EUV-probe measurements of 1 fs scale dynamics in atoms^9^,^10^ and molecules^18^. In this work we demonstrate an advanced instrument for quantitative measurements in the EUV spectral region that is based on the spatially resolved measurement of ionization products that it provides.

Quantities closely related to non-linear EUV ionization are the generalized cross sections of multi-EUV-photon processes. Cross sections of multiphoton ionization processes have been successfully measured in the past at both the IR and UV spectral regions. Most of them rely on the saturation of multiphoton ionization^19^,^20^,^21^,^22^ and thus are hardly applicable to the EUV spectral range due to the limited number of sources able to cause saturation of the ionization. To our knowledge, there is only one measurement of the generalized two-EUV-photon ionization cross section (σ^(2)^) of Helium in the 20 eV photon energy range. This measurement was performed using high intensity tunable EUV radiation delivered by a free electron laser (FEL) source^23^. Here, we demonstrate an approach with which the measurement of the two-EUV-photon ionization cross section of Helium can be performed over a large intensity range using the image of the Helium ion distribution, which is produced by a table top EUV source. The successful measurement of the generalized cross section demonstrates the potential of the approach in performing non-linear spectroscopic studies at EUV spectral range and provides experimental input to test the validity of the relevant theoretical models.

For both EUV pulse metrology as well as for pump-probe investigations it is highly desirable to have a fast pulse duration characterization method. This is particularly important when non-linear processes are observed using driving lasers with poor energy, carrier envelope (CEP), or duration stability. Then, single-shot or at the most a few-shot measurement of the pulse duration is required in order to apply tagging approaches^24^,^25^ in the measured spectra or in order to easily optimize or change parameters. In a recent study we have proposed and modeled a novel single-shot second-order autocorrelation scheme for EUV radiation^26^. As in common optical 2^nd^ order single-shot autocorrelators, what is needed to be recorded is the spatially resolved pattern of the products of the second order process induced by two crossed beams of the radiation to be characterized. The temporal profile of the radiation is then mapped to the measured spatial distribution.

In attosecond metrology, two-photon processes, such as second harmonic generation in crystals, as used in fs pulse metrology, has been replaced by two-photon ionization in the EUV spectral region^9^,^10^,^16^,^17^. Observable two-EUV-photon ionization for a single shot autocorrelation measurement, is today straightforward and feasible using multi-cycle high peak power driving laser pulses and loose focusing geometries in high harmonic generation in gas media. However, spatially resolved distributions of the two-EUV-photon ionization have not yet been demonstrated. Spatially resolved ionization produced at the focus of a harmonic comb through single photon ionization of Argon atoms has been recently achieved using a high spatial resolution ion microscope^27^. In ref. 27, the measured spatial distributions have revealed interference effects originating from the long and short electron trajectories participating in the harmonic generation process. In this work, we further report the first observation of spatially resolved two-EUV-photon ionization of atoms by a comb of higher order harmonics. This is a further step towards single-shot non-linear EUV autocorrelation measurements. However, for the final goal of a single shot measurement further important, far from trivial steps, have to be made^26^.

## Experimental procedure

**Figure 1:**
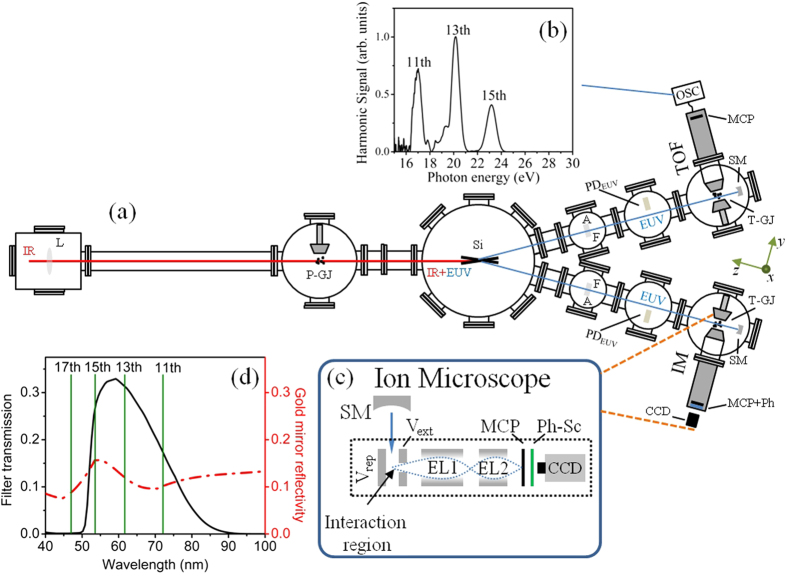
Experimental set-up. (**a**) Experimental set-up. The *y*-axis is parallel to the TOF axis and the *x*-axis is parallel to the plane of the detector (MCP + Ph). (**b**) The spectrum of the harmonics used in the I-ID branch. (**c**) A drawing of the Ion Microscope. (**d**) EUV transmission curve of a 150 nm thick Sn filter (black line) and reflectivity of the gold mirror (red dashed line).

The experimental setup used is shown in Fig. 1a. A 10 Hz repetition rate Ti:Sapphire laser system delivers pulses of up to 170 mJ energy, τ_*L*_ = 33 fs duration and wavelength of 800 nm (IR). An annular laser beam (formed using a super-Gaussian beam stop of 5 mm diameter, deposited on a BK7 plate) with an outer diameter of ≈2.5 cm and energy of ≈15 mJ/pulse is focused with an *f* = 3 m focal length lens into a pulsed gas jet (P-GJ) filled with Xe, where the harmonic radiation is generated. We use an annular IR beam because after the focus the IR beam remains annular while the EUV radiation is propagating on axis in the cone at the center of the annular beam, where no IR radiation is propagating. Thus, by using an aperture of appropriate diameter the IR beam can be blocked, while the EUV radiation is propagating though it, i.e. the combination of an annular beam with an aperture facilitates the elimination of the IR radiation. The super Gaussian beam stop is used in order to avoid IR diffraction fringes co-propagating with the EUV beam. A Si plate, placed after the jet at the Brewster angle for the fundamental (75°), reflects the harmonics^28^ towards the detection area, while substantially attenuating the IR field. After reflection from the Si plate, the EUV radiation passes through a 5 mm diameter aperture (A) which blocks the residual outer part of the IR beam. A 150 nm thick Sn filter (F) selects the 11th to 15th harmonics with approximately equal amplitudes. Subsequently, the EUV beam is focused into the target Helium gas jet (T-GJ) with a spherical gold mirror (SM) of 5 cm focal length. The characteristics of both nozzles used can be found in the literature^29^. Two-photon ionization of Helium occurs from the 11^th^ to 15^th^ harmonics. The harmonic spectrum measured by recording energy resolved photoelectron spectra resulting from the single-photon photoionization of Ar by the harmonic comb, is shown in Fig. 1b. The electron spectra were recorded using a μ-metal shielded time-of-flight (TOF) ion/electron spectrometer, attached to a second EUV beam-line branch (upper branch in Fig. 1a). Ions are measured using an Ion Microscope (IM)^30^ (Fig. 1c) that images the focal area onto a Micro-channel Plate (MCP) detector equipped with a phosphor (Ph) screen anode. A CCD camera records the images of the phosphor screen and stores them on a PC. The resolution of the IM is ≈ 1*μm*. The interaction region of the IM is filled with the gas under investigation (Ar or He) by means of a piezo pulsed nozzle working with backing pressures of ≈3 bar and ≈1 bar for Ar and He, respectively. The body of the nozzle was floated at a voltage of 5 kV (V_rep_ in Fig. 1c). The ion distribution in the EUV focus (located in the object plane) is mapped onto a position-sensitive detector located in the image plane and reveals their spatial distribution. The ions that are generated in the EUV focus are first accelerated by an electric field applied between the repeller and extractor electrodes (V_ext_ ≈ 4.5 kV). The first electrostatic lens (EL1 in Fig. 1c) images the spatial extent of the ion cloud into the ion microscope with a small magnification factor. This intermediate image is located in the focal plane of a second electrostatic lens (EL2 in Fig. 1c) that projects a further magnified image onto the detector consisting of a pair of MCPs and a phosphor screen. The voltages applied on EL1 and EL2 were ≈2.5 kV and ≈3.5 kV, respectively. The voltages applied on the IM strongly depend on the position of the focus in the acceleration region. In the present experiment they have been set such that a sufficient for the experiment magnification (≈80) is achieved and the field of view is made comparable with the length of the EUV focus. It should be noted here that the magnification of the IM used in this experiment, was different than that in ref. 30 and thus the field of view differs in the two works. The image of the ion cloud that appears on the phosphor screen is recorded with a CCD camera. Since ions with different charge to mass ratios have different flight times, gating the MCP detector with a time window of ≈80 ns enables us to select individual charge to mass states. The structural resolution of the EUV focus is inversed proportional to the ion density, the square of the EUV radius and depends on the DC electric field strengths applied in the IM. This is due to the repulsive forces among the ions (space charge effects) at the point of the generation until the image plane. The effect of the repulsive forces can be suppressed either by reducing the density of ions or by increasing the electric field strengths applied in the IM. In order to have the same experimental conditions in both the TOF and the IM set-ups, the TOF branch was constructed in an identical way to the IM. Thus the two symmetric branches in Fig. 1a are used for different diagnostics, i.e. for measuring energy resolved photoelectron spectra resulting from the interaction of the EUV with gas targets (upper branch) or the spatially resolved ion distribution resulting from the interaction of the EUV with gas targets (lower branch). Any two-color (IR-EUV) two- photon ionization process can be excluded due to the elimination of the IR radiation in the detection region. This is verified by separate measurements of the energy resolved single-EUV-photon ionization photoelectron spectrum of Ar in which “side bands” are entirely absent. It is worth noting that in the photon energy range between 15 eV and 30 eV the single-photon-ionization cross section of Argon is varying only from 30 to 35 Mb^31^,^32^,^33^. Thus, the measured photoelectron spectral distribution does not differ significantly from the EUV spectral distribution. The energy of the EUV radiation in the interaction region of the IM was obtained from the measured pulse energy using an EUV calibrated photodiode (PD_EUV_) taking into account the reflectivity of the gold spherical mirror (Fig. 1d). The PD_EUV_ has been placed after the aperture (A) and the filter (F).

## Results

**Figure 2:**
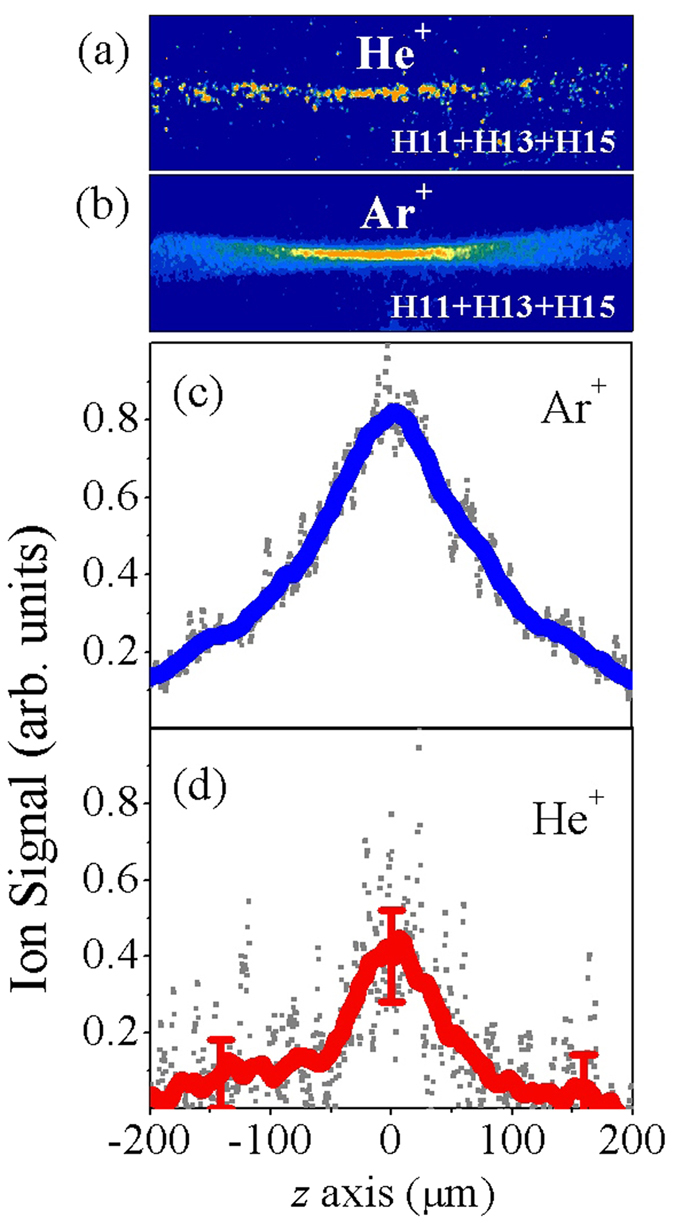
Spatial ion distributions at the EUV focus induced by single- and two- photon ionization. He^+^ (**a**) and Ar^+^ (**b**) ion distributions at the EUV focus. For Ar^+^ and He^+^ images 600 and 15000 shots were accumulated, respectively. Both images have been obtained after the subtraction of background images which were recorded having the pulsed nozzle in the IM region closed. (**c,d**) Ion signal along the propagation axis (line-out) at the centre of the Ar^+^ and He^+^ distributions. The blue and red lines are the 60-point running average of the raw data (gray dots). In (**b,c**) the thickness of the line and the error bars represent one standard deviation of the mean values.

An EUV pulse with duration *τ*_*EUV*_ ≈ 15 fs, energy 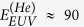 nJ/pulse and peak intensity ≈10^14^ W/cm^2^ in the interaction area was used for the measurement of the EUV image resulting from the two-EUV-photon ionization of He. The pulse duration of the EUV radiation has been estimated using the relation 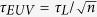 (where *τ*_*L*_ is the duration of the laser pulse and *n* = 3–5 is the effective order of non-linearity of the harmonic generation process close to the plateau region^34^). The peak intensity of the EUV pulse in the interaction region was found using the EUV pulse energy of ≈ 90 nJ, the estimated pulse duration of 15 fs and the measured focal spot diameter of 4μm taking into account the reflectivity of the gold coated mirror (Fig. 1d) up to the 15^th^ harmonic. Fig. 2a depicts the spatial He^+^ ion distribution in the EUV focal area that was measured for a laser intensity of ≈10^14^ W/cm^2^ in the harmonic generation area. This image has been compared with the ion distribution produced by single photon ionization of Argon (Fig. 2b). The images are the projections of the three dimensional ion distributions in the observation plane. The Ar^+^ distribution was recorded using a reduced EUV energy of 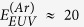 nJ/pulse in the interaction region. The EUV energy was reduced by decreasing the Xenon pressure in the EUV generation area. It has been verified by measuring harmonic spectra at different Xenon pressures, that the EUV energy lowering through the reduction of the pressure does not affect the structure of the harmonic spectrum. The reduction of the EUV energy was done in order to minimize the influence of space charge effects in the Ar^+^ image. This is the only change made when comparing the Ar^+^ and He^+^ images. This has been achieved by changing only the gas in the T-GJ nozzle. In this way the Ar^+^ distribution was generated at the same place in the interaction region as the He^+^ distribution. Figure 2c,d depict the Argon and Helium ion distributions, respectively, along the propagation axis (*z*) at the centre (*x* = 0) of the ion distribution. In the two-photon-He-ionization case a lower signal and narrower spatial distribution are expected due to the non-linearity of the ionization process. Indeed, comparing the measured Helium image with those of the single-EUV-photon ionization of Argon, we can see that i) the noise of the image of Fig. 2a is greater than that of image Fig. 2b and ii) the width of the spatial distributions in Fig. 2a,d is narrower by a factor of ≈1.4 compared to those in Fig. 2b,c. This is compatible with a two-photon ionization process. These two features verify that the observed ion distributions are produced through two-photon-ionization of Helium as expected for the ionizing harmonic photon energies. Furthermore, taking into account the transmission of the Sn filter, the reflectivity of the Gold spherical mirror and the harmonic spectrum generated by the Xe gas, it has been found that the contribution of the single-photon ionization of He resulted in harmonics with photon energies >25 eV is more than an order of magnitude lower compared to the two-photon ionization signal and thus can be considered negligible. This further supports the dominance of the two-EUV-photon ionization process.

**Figure 3:**
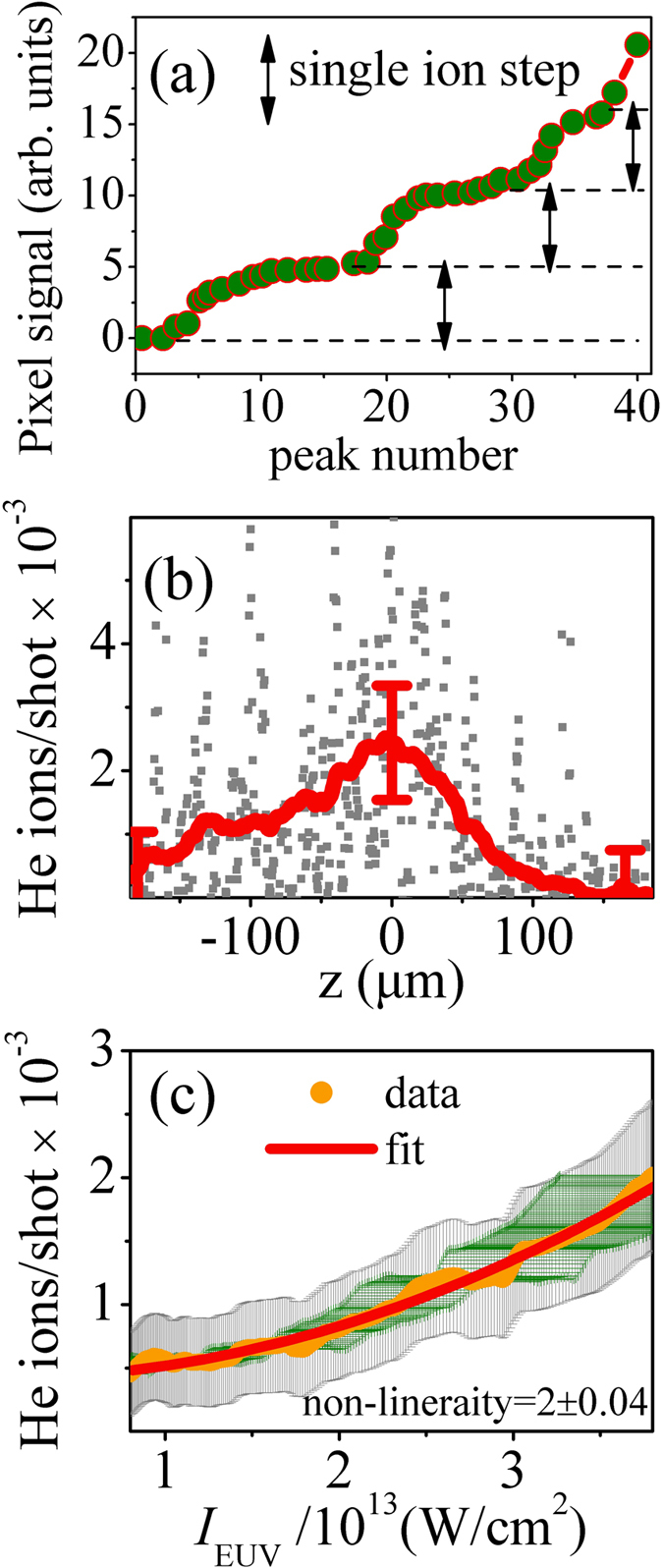
Dependence of the two-photon He^+^ yield on the EUV intensity. (**a**) Calibration of the I-ID detector on the single ion detection. The *y*-axis shows the signal height of individual pixels. The *x*-axis is the peak number where numbering is such that the peak height increases with increasing peak number. The background noise level corresponds to a value of ±0.6 while the single ion count correspond to the value of ≈5. (**b**) Number of generated He^+^ per shot in the EUV focus area. The red line is the 170-point running average of the raw data (gray dots) and the error bars represent one standard deviation of the mean value. (**c**) Dependence of the number of He^+^ per shot per *δz* on the intensity of the EUV radiation (orange dots). The dependence has been obtained using the values of the red line of (**b**). The red line shows the non-linear fit of the data. The gray and green error bars in the number of He^+^ per shot and in EUV intensity, respectively, represent one standard deviation of the mean value.

**Figure 4:**
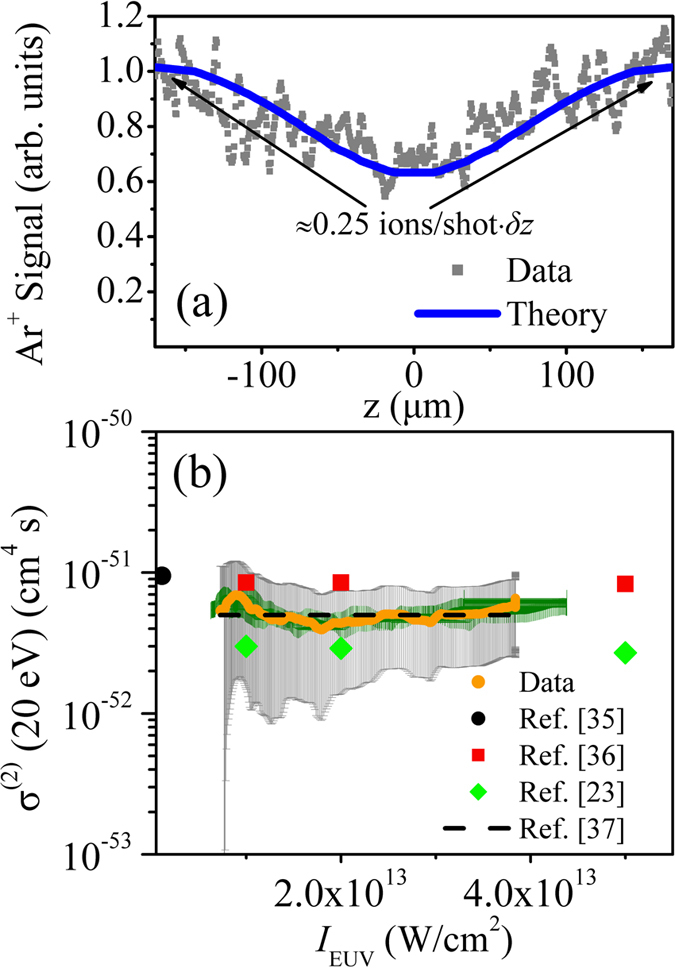
Quantitative measurements of the generalized cross sections of the one- and two-EUV-photon ionization process. (**a**) Volume integrated Ar^+^ signal (normalized to unit value) at each position of the EUV focus (gray points). The blue solid line shows the theoretically calculated Ar^+^ signal. (**b**) Measurement of the σ^(2)^ of Helium (orange points) at the ≈7 × 10^12^ W/cm^2^ to ≈4 × 10^13^ W/cm^2^ EUV intensity range for EUV radiation of carrier wavelength 61.5 nm (photon energy = 20 eV). The gray and green error bars in cross section and in EUV intensity, respectively, represent one standard deviation of the mean value. The black-filled circles and the red-filled squares are the theoretical cross section values taken from refs 35,36, respectively. The black dashed line represents the σ^(2)^ value which is calculated according to eq. 15 of ref. 37. The green-filled diamonds are the experimentally measured cross section values taken from ref. 23.

The non-linearity of the ionization process of Helium was directly confirmed by measuring the dependence of the Helium ion yield on the intensity of the EUV radiation. By calibrating the detector for single ion detection (Fig. 3a), the number of the generated Helium ions/shot (integrated signal along the *x*-axis) was obtained for each position (*z*) in the EUV focus area (Fig. 3b). The intensity of the EUV radiation at each position of the propagation axis was obtained using the relation 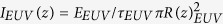 (where *R*_*EUV*_(*z*) is the radius of the EUV beam at each position of the propagation axis). The *R*_*EUV*_(*z*) values were obtained from the Ar^+^ image. The dependence of the number of Helium ions/shot (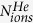) on the intensity of the EUV radiation is shown in Fig. 3c. The power of the non-linear fitted function (red solid line of Fig. 3c) was found to be ≈2, which is in agreement with Fig. 2d. According to Lowest Order Perturbation Theory (LOPT) and using the data of Fig. 3c, the generalized two-EUV-photon ionization cross section of Helium was obtained for a relatively large intensity range (Fig. 4) using the relation 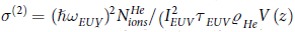). *ρ*_*He*_ is the density of Helium atoms in the interaction region and 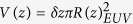 is the interaction volume (where *δz* = 0.52 μm). Since the Ar^+^ image was recorded under the same experimental geometry as the He^+^ image it can be assumed that *ρ*_*He*_≈ *ρ*_*Ar*_. Thus, the density *ρ*_*He*_ can be obtained using the number of Ar^+^ ions/shot which was obtained from the Ar^+^ image at *z* ≈ ± 160 μm. Figure 4a shows the number of generated Ar^+^ ions/shot (integrated signal over the *x*-axis) at each position (*z*) in the EUV focus area (gray points). The reduction of the signal for *z* positions closer to the focus is attributed to the saturation effects of Ar single-photon ionization. This has been confirmed by the theoretical calculations (blue solid line in Fig. 4a) which are found to be in agreement with the experimental findings. This curve was calculated using the relation^19^,^20^,^21^,^22^ 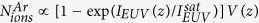 with 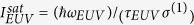. From the intensity values (*I*_*EUV*_(*z*)) already obtained in the present work, we find a single-photon ionization cross section *σ*^(1)^ ≈33 Mb which is in agreement with previously measured values^31^,^32^,^33^. Alternatively, using the single-photon ionization cross section known from the literature, Fig. 4a can be used for the accurate measurement of 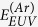 in the interaction area. Thus, the obtained 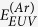 at the interaction area is found to be in agreement with the result of the calibrated EUV photodiode i.e. ≈20 nJ/pulse. This self calibration procedure is an advantage of the present method and ensures an accurate measurement of the generalized cross section values in the EUV spectral region. Based on LOPT for the single-EUV-photon ionization of Argon, 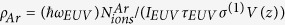 (where *σ*^(1)^ ≈ 33 Mb at ≈20 eV photon energy) was found to be *ρ*_*He*_ ≈ 2 × 10^11^ atoms/cm^3^. In the 7 × 10^12^ W/cm^2^ − 4 × 10^13^ W/cm^2^ intensity range the generalized two-EUV-photon ionization cross section of Helium is found to be *σ*^(2)^ ≈ 5 × 10^−52^ cm^4^s which is in reasonable agreement with the theoretical^35^,^36^,^37^ and experimental^23^ values. The black dashed line in Fig. 4b shows *σ*^(2)^ of Helium which was calculated using the equation 
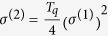
 (where *T*_q_ is the period of the 13th harmonic and *σ*^(1)^ ≈ 3 × 10^−18^ cm^2^ is the single-photon ionization of Helium for EUV photon energy of 2 × *ħ*ω_q_ = 40 eV) of ref. 37.

## Discussion

It is worth noting, that the amount of the signal can be significantly enhanced, while the number of shots and the error of the measurements can be significantly reduced by optimizing the conditions in the IM interaction area. This can be done by increasing the density of Helium atoms and the acceleration voltages in the IM interaction region^38^. Under these conditions a single-shot He^+^ image, which is required for a single-shot autocorrelation measurement, could be recorded. What remains to be realized for the single-shot non-linear autocorrelation measurement is the implementation of the experiment using two EUV beam replicas crossed at a small angle in the ionization focal area. Experimental set-ups and detailed information about the requirements for developing a single-shot-XUV-autocorreletor are presented elsewhere^26^. Of course, the enhancement of the energy of the EUV radiation is an additional factor which can significantly contribute to the improvement of the measurements and the realization of a single-shot 2^nd^ order autocorrelator.

## Conclusions

In conclusion, using the ion microscope device, we have demonstrated a self- calibrating method for measuring cross-sections of linear- and non-linear ionization processes in the EUV range. The experimental findings achieved using this device are precursors to a single-shot 2^nd^ order autocorrelation in the EUV range. The above results have been achieved by spatially resolving the products of the EUV-ionization of atoms induced by energetic high order harmonic radiation pulses.

## Additional Information

**How to cite this article**: Tsatrafyllis, N. *et al*. The ion microscope as a tool for quantitative measurements in the extreme ultraviolet. *Sci. Rep.* **6**, 21556; doi: 10.1038/srep21556 (2016).
